# Calomel and Soda as a Cathartic

**Published:** 1852-06

**Authors:** H. Hunt

**Affiliations:** Delevan, Wisconsin


From the Boston Med. and Surg. Jour.

*Calomel and Soda as a Cathartic.* By Dr. H. Hunt, of Delevan, Wisconsin.

The first time I used this, or knew of its being used, was four years ago last December, in my own case, while in the city of New York. I had been living and practising in the miasmatic West for the previous eleven years; and although I had never had an attack of fever, still my system was more or less debilitated, and my liver and bowels quite torpid. For this I consulted Prof. Dickson, who advised me to take one grain of calomel at bed-time for a number of nights in succession, and drink an infusion of Peruvian bark. Preferring to take the calomel in the form of pill, I united it with six or eight grains of bicarb. soda, and formed into pills by hard soap. I took this at 10 o'clock, P. M., and although my bowels had been thoroughly constipated for a number of days, I had a thorough operation by seven in the morning, and some three more followed in quick succession. At first I attributed the movements to the setting in of a diarrhœa following constipation; but by using it in a few days again in my case, as well as in that of others, I found the same effects to follow its administration as in the first instance, though not quite so thorough. I had been using soda as an antacid freely, but without any cathartic tendency whatever, and in uniting it with the one grain of calomel, my object was to give bulk, and also to neutralize acid in my stomach, with which I had been very much troubled. When I returned home in the spring, I had the most satisfactory demonstration of its efficacy as an anti-bilious purge, for there were some old cases of habitual tendency to attacks of torpor of liver and bowels that had troubled me exceedingly to physic. There was one man in particular whom I had treated for this trouble, and whom I had given within twenty-four hours forty grains of calomel, as much jalap, near half a pound, of salts, a large quantity of castor oil, injections of jalap and senna, &c. And after all this mighty array of cathartics and injections, still the result was a trifling purgation, and the patient *gradually* recovered. These cases were easily operated on by the use of three or four grs. of cal., and from ten to twenty grs. of soda. This dose was all that was necessary to purge the case alluded to above, "to his heart's content," and in such cases it has never failed to do the business promptly and thoroughly.

In a common case, I give two grs. cal., well levigated with from ten to twenty grs. of bicarb. soda in molasses. This will almost always operate by morning, if given at bedtime. Dr. Bradway unites them in the proportion of one cal. to three of soda; but I have generally united them in the proportion of one cal. to five of soda.

After the liver and bowels have been thoroughly operated on, the medicine has much less effect, and I therefore desist for a few days, or entirely, for the obvious reason that the important indications are fulfilled.

